# Feasibility, Acceptability, and Preliminary Outcomes of an Integrated Telemedicine Intervention Combining Naltrexone and Cognitive Behavioral Therapy for Alcohol Use Disorder

**DOI:** 10.1089/tmr.2022.0029

**Published:** 2022-11-04

**Authors:** Suzette Glasner, Jamie Webb, Darcy Michero, Courtney Motschman, Laura Monico, Alfonso Ang, Peyton Pielsticker

**Affiliations:** ^1^ Department of Clinical Affairs, Digital Therapeutics, Inc., San Francisco, California, USA.; ^2^ Department of Psychiatry & Biobehavioral Sciences, UCLA Integrated Substance Abuse Programs, Los Angeles, California, USA.; ^3^ Department of Psychological Sciences, University of Missouri, Columbia, Missouri, USA.; ^4^ Friends Research Institute, Baltimore, Maryland, USA.

**Keywords:** cognitive behavioral therapy, alcohol use disorder, naltrexone, m-health

## Abstract

**Background:**

A small fraction of individuals in need of treatment for alcohol use disorders (AUDs) seek care, owing largely to barriers to accessing treatment. In the present pilot study, we examine the feasibility, acceptability, and preliminary outcomes of an m-health intervention combining cognitive behavioral therapy and pharmacotherapy for individuals with AUD.

**Methods:**

Adults with AUD (*N* = 26) recruited through online, social media-based advertising were enrolled in a 12-week, integrated telemedicine intervention combining psychosocial treatment with medical management: Quit Genius for AUD (QG-A). Feasibility, acceptability, perceived helpfulness, treatment engagement, retention, completion, and clinical outcomes including alcohol use and secondary mental health outcomes were assessed.

**Results:**

Participants found the QG-A intervention to be acceptable and helpful in facilitating action toward their therapeutic goals concerning alcohol use. Treatment completion, achieved by the majority (85%) of participants, was excellent. On average, participants reduced their past 30-day alcohol use from baseline (mean proportion of days of abstinent = 0.13) to follow-up (*M* = 0.59), *t*(19) = −4.97, *p* < 0.001, and consumed fewer drinks per drinking day from baseline (*M* = 6.7) to follow-up (*M* = 2.0), *t*(19) = 3.61, *p* < 0.001. Concurrently, reductions were observed in depressive (*t*[22] = 5.39, *p* < 0.001) and anxiety (*t*[22] = 2.87, *p* < 0.01) symptom severity, from the moderately severe range at baseline to the mild range at treatment-end, with increases in resilience (*t*[22] = −3.54, *p* < 0.001).

**Conclusions:**

Addressing AUDs using an integrated m-health intervention to deliver evidence-based psychosocial and pharmacological treatment is feasible and may produce improvements in both alcohol use and psychiatric symptoms.

## Introduction

Alcohol-related problems account for 3 million deaths annually, making alcohol use disorder (AUD) a major global cause of preventable morbidity and mortality.^1^ Since the onset of the COVID-19 pandemic, alcohol consumption increased by 25%, which, if sustained for even 1 year, is estimated to cause over 18,000 new cases of liver failure and 8000 additional deaths from liver disease.^2^

Pharmacotherapy in combination with behavioral treatment is efficacious for AUD, and integration of these approaches is considered best practice in addiction treatment.^3^ In particular, cognitive behavioral therapy (CBT) and motivational enhancement therapy (MET) have been highly successful when used in combination with the opioid receptor antagonist naltrexone (NTX).^4^ Despite efficacious treatment options for AUD, fewer than 10% of U.S. individuals with AUD received treatment within the past year, and only 4% received an Food and Drug Administration (FDA)-approved AUD medication.^5^ Barriers to seeking treatment include limited accessibility of evidence-based care, concerns about stigmatization, low motivation, and cost of treatment.^6,7^

The practice of telemedicine, particularly when delivered through m-health technology, confers many advantages over traditional treatment modalities, including convenience, cost-effectiveness, and privacy.^8^ Importantly, incorporating human contact as a component of technology-based interventions produces superior engagement and outcomes among those with AUD, relative to self-guided fully asynchronous approaches.^9^

Despite a growing number of m-health interventions targeting AUD, the majority serve as platforms for either behavioral treatment or medication management (MM), leaving an opportunity for a high-quality app using combination treatments.^10–12^ Moreover, despite the acceleration of telehealth services targeting opioid use disorders during the COVID-19 pandemic,^13,14^ relatively few innovations in telemedicine approaches to the treatment of AUD have emerged.^15^

In the present study, we evaluate the feasibility, acceptability, and preliminary outcomes of a novel integrated telemedicine intervention for AUD, utilizing a combination of an m-health app accessible via smartphone, clinician-delivered behavioral therapy, and pharmacotherapy. We hypothesized that adults with AUD would find the intervention to be helpful in achieving their treatment goals, and participation would be associated with reductions in alcohol use and associated psychiatric symptoms.

## Methods

### Participants

Participants were 26 adults with AUD. The study was approved by Ethical and Independent Review Services' institutional review board. Participants were recruited from social media (i.e., Facebook) advertisements and directed to a study website, where a description of the study and pre-screening questions were provided. Potential participants were invited to complete a telephonic assessment with a research counselor to confirm eligibility.

To be eligible, participants were required to: (1) be ≥18 years old; (2) reside in the United States; (3) own a smartphone with sufficient functionality to download and utilize the study m-health app; (4) have a Diagnostic and Statistical Manual of Mental Disorders, Fifth Edition (DSM-5) diagnosis of AUD; (5) be able and willing to participate in study procedures, including taking study medication (NTX); and (6) be in good general health. Individuals were excluded if they: (1) had a known sensitivity to NTX; (2) had a serious medical condition that would make participation hazardous; (3) required medical detoxification from any substances; (4) had used acamprosate, disulfiram, or NTX, within the 30 days before screening; (5) were routinely taking opioid medication or anticipated surgery that would require opioid maintenance during the study; (6) had undergone medical detoxification more than once; (7) lacked English proficiency; or (8) had clinically significant psychiatric symptoms that would make study participation difficult. The AUD diagnoses were assessed using the DSM-5 checklist.^16^

Following a complete description of the study to participants, informed consent was obtained. Participants agreed to: (1) Twelve weekly 45-min video-based, counselor-delivered CBT sessions; (2) Three monthly virtual MM visits with a study provider; (3) monthly online self-report assessments, with $40 compensation for the baseline, and $25 for each of the monthly assessments.

### Procedure

#### Design

Eligible participants completed a baseline diagnostic assessment with a study counselor, based on the American Society of Addiction Medicine's guidelines^17^ and a biopsychosocial assessment with a study physician or nurse practitioner licensed in the participants' state of residence to confirm eligibility; for continuity, this clinician was assigned to the participant for MM for the duration of the study. Subsequently, participants were inducted onto NTX and scheduled for weekly counseling visits and monthly MM visits over 12 weeks.

At each counseling visit, participants received manualized, counselor-delivered CBT. At each MM visit, dosage, side effects, and treatment progress were reviewed and adjusted as clinically indicated by a study clinician. Participants also completed monthly research assessments. At treatment-end, nine randomly selected participants were interviewed individually online about their experience with the intervention.

### Intervention

**FIG. 1.**
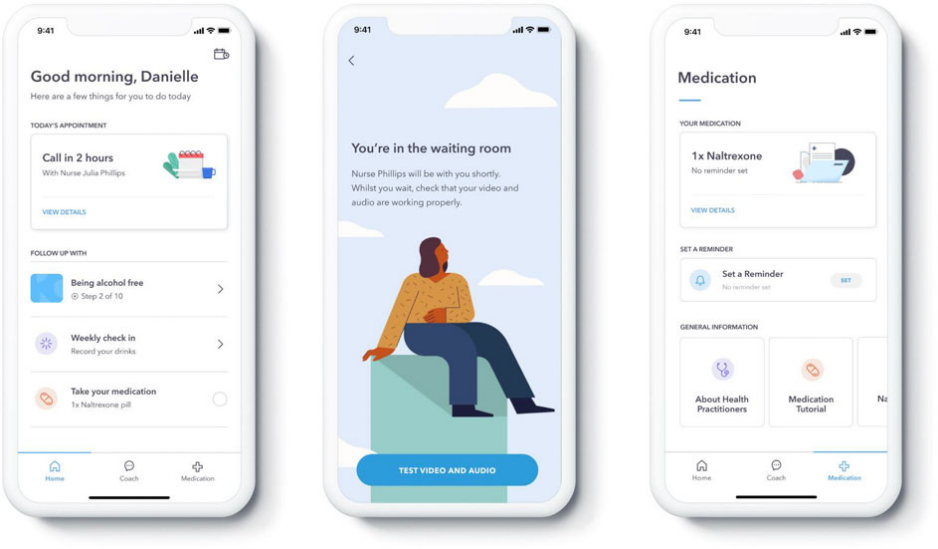
Screenshots of the QG-A m-health intervention. QG-A, Quit Genius for AUD.

Quit Genius for AUD (QG-A), a commercially available outpatient treatment program (Digital Therapeutics, Inc., San Francisco, CA), comprises a 12-week, integrated digital intervention combining pharmacotherapy and CBT/MET for AUD (Fig. 1). The app platform from which the intervention is delivered includes a Health Insurance Portability and Accountability Act of 1996 compliant and the Health Information Trust Alliance certified videoconferencing platform for virtual counseling and MM visits, digital CBT/MET skills training content in interactive modules,^18^ and a chat function for asynchronous messaging with the counselor.

The text in the digital CBT/MET content is at the sixth grade reading level and has audio accompaniment. Self-reported alcohol use quantity and frequency is gathered through the app on a weekly basis.

QG-A allows for a range of therapeutic objectives, from reduction of drinking to total abstinence. App content includes modules on bolstering motivation to change, understanding triggers, behavioral and cognitive strategies for managing cravings, problem solving, alcohol refusal skills, establishing social support, and increasing pleasurable activities. Therapeutic content in the app was delivered via a combination of text, bidirectional messaging, interactive skills training exercises, and tailored motivational content to reinforce personalized health and cost-savings achievements.

Counseling was provided by master's level study therapists who were formally trained before the present study in CBT and MET, and received standardized training and weekly group and individual clinical supervision from the Principal Investigator.

#### Medication management

After confirming eligibility for pharmacotherapy, participants were inducted onto NTX by a study physician or nurse practitioner according to standard clinical practice. NTX was titrated to 50 mg/day. Participants attended monthly, 15-min videoconferencing-based MM visits, comprising dose adjustments and limited “counseling” as normally provided to patients in outpatient settings. Information and answers to commonly asked questions concerning NTX was accessible within the QG-A app. Four weeks before the end of treatment, participants were either given instructions to discontinue NTX under the study physician's supervision or referred to a provider for continued treatment.

### Measures

A trained research assistant conducted assessments by phone at baseline and monthly over the 12-week treatment phase. Self-report questionnaires were completed by participants electronically using a link that was emailed to them. AUD was diagnosed at baseline using the DSM-5 checklist.^16^

#### Alcohol use

Alcohol use during the past 30 days was assessed at baseline and monthly thereafter using the Timeline Follow Back, a calendar-assisted structured interview.^19^ Primary outcomes were proportion of days abstinent and drinks per drinking day.^20^ Changes in the World Health Organization (WHO) drinking risk level from baseline to treatment-end were examined as a secondary outcome.

#### Negative affect

Changes in negative affect were examined at baseline and monthly thereafter using the Patient Health Questionnaire depression scale and the Generalized Anxiety Disorder 7-item scale.^21,22^

#### Resilience

Given the association of stress with increased alcohol use during COVID-19, we examined changes in resilience, or the ability to bounce back and recover from stress, over the course of treatment. The Brief Resilience Scale, a reliable and valid 6-item self-report measure, was administered at baseline and monthly thereafter.^23^

#### Statistical analysis

Alcohol use in the past 30 days was indicated by: (1) proportion of days abstinent; (2) number of standard drinks per drinking day. Chi-square and *t*-tests were used to analyze baseline to post-treatment differences on alcohol use and secondary outcomes.

## Results

Over the 9-month study period, 83 individuals were screened, of whom 65 consented to participate. Of those, 26 individuals were inducted onto NTX and given the m-health intervention. Of the 65 consented participants, 39 failed or did not complete the full screen for inclusion/exclusion criteria following consent. The three study therapists were each assigned nine participants, on average (standard deviation [SD] = 6), and completed a mean of 8.9 sessions per assigned participant (range = 1–12, SD = 3.6). On average, participants were 45 years of age (SD = 13), predominantly male (71%), employed (67%), high school or college educated (71%), and Caucasian (68%; Table 1). At baseline, participants reported having consumed alcohol on 25.9 of the past 30 days (SD = 5.8), with an average of 6.7 drinks per drinking day (SD = 3.8).

**Table 1.** Sample Demographic Characteristics

Characteristic	Sample (***N*** = 26)
Age, mean (SD)	44.4 (13.2)
Gender, *n* (%)	
Male	18 (69.2)
Female	8 (30.8)
Education, *n* (%)	
High school or less	10 (38.4)
Bachelor's degree	9 (34.6)
Graduate degree (masters, JD, PhD)	7 (27.0)
Race, *n* (%)	
White	17 (65.4)
African-American	5 (19.2)
Hispanic	3 (11.5)
Native American	1 (3.9)
Employment, *n* (%)	
Employed	17 (65.4)
Not employed	9 (34.6)

SD, standard deviation.

Treatment acceptability, defined among those who were inducted on to NTX and given the m-health intervention as attending at least 2 data collection visits (4+ weeks of study participation), was excellent (96%). On average, participants attended 9 of the 12 counselor-facilitated CBT sessions (SD = 3, range = 1–12). Treatment completion, defined as attending the week 12 data collection session, was achieved by the majority of participants (85%).

**FIG. 2.**
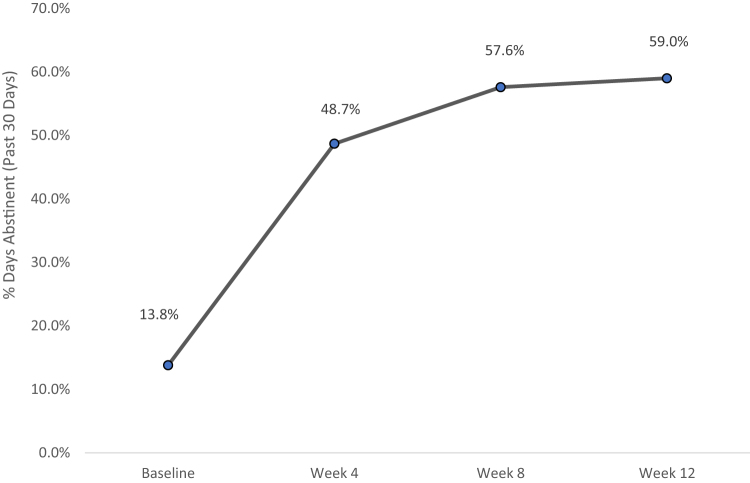
Changes in mean percentage of days abstinent from baseline to week 12.

**FIG. 3.**
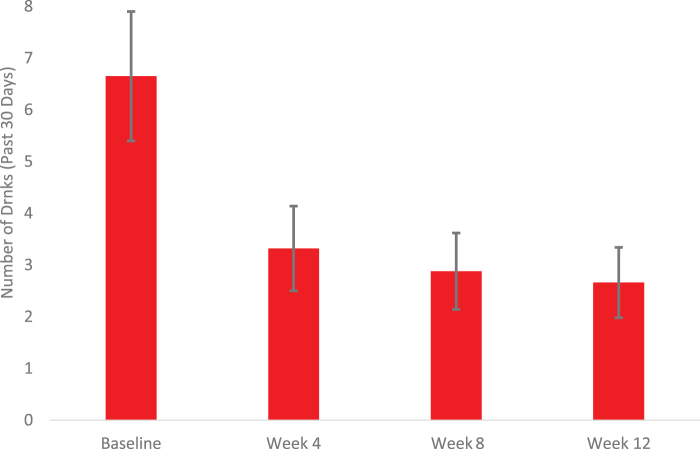
Changes in mean drinks per drinking day from baseline to week 12.

The proportion of days abstinent in the past 30 increased from baseline (*M* = 0.13, SD = 0.19) to treatment-end (*M* = 0.59, SD = 0.30), *t*(19) = −4.9, *p* < 0.001 (Fig. 2). Likewise, drinks per drinking day declined from baseline to discharge (*M* = 6.7, SD = 3.8 vs. *M* = 2.7, SD = 2.0), *t*(19) = 3.6, *p* < 0.0.001 (Fig. 3). Over two-thirds of participants (68%) reduced their alcohol use from baseline to treatment-end by one or more WHO risk drinking levels (*χ*^2^ = 15.2, *p* < 0.01), with 26% having reduced by 1 level and 42% reducing by 2 or 3 levels.

Depressive symptoms reduced from the moderately severe range at baseline (*M* = 8.3, SD = 5.0) to the mild range at week 12 (*M* = 4.1, SD = 3.5), *t*(22) = 5.4, *p* < 0.001. Likewise, anxiety severity declined from the moderate (*M* = 7.1, SD = 6.7) to the mild (*M* = 4.7, SD = 6.1) range *t*(22) = 2.9, *p* < 0.01. Resilience increased over the course of treatment (*M* = 3.1 vs. *M* = 3.7), *t*(22) = −3.5, *p* < 0.001.

On average, participants remained engaged in the QG-A intervention for 10.3 weeks (SD = 2.8; Table 2). The odds of achieving a “good clinical outcome,” defined as proportion of days abstinent ≥0.75 in the 30 days preceding treatment-end, were greater among those who remained engaged with QG-A for ≥9 weeks (odds ratio = 3.2, *p* = 0.03) compared with those who engaged for 8 weeks or less. Perceived helpfulness of QG-A was assessed using a consumer feedback questionnaire.

**Table 2.** Indicators of Quit Genius for Alcohol Use Disorder Intervention Engagement

Engagement metric	Mean (SD)	Range
Number of weeks engaged with QG-A	10.3 (2.8)	2–12
Number of times app was opened	75.4 (56.4)	9–240
Total minutes spent on app	283.5 (167.6)	35–680
Total chat messages sent to counselor	22.2 (16.3)	1–54
Total alcohol use check-ins	7.5 (5.2)	0–16

QG-A, Quit Genius for AUD.

The majority of participants agreed or strongly agreed that QG-A helped them change their alcohol use (83%) and would recommend QG-A to others (95%). Qualitative feedback concerning QG-A included the following: “I cut back on alcohol consumption a lot…[my counselor] encouraged me to engage in alternatives like physical exercise…my health has improved dramatically.” With the counselor, “I felt very safe. I felt very supported…the relationship was very positive…I also enjoyed the [app] content.” Regarding pharmacotherapy, “I had one drink and then…didn't have another one, which has never happened in my life…I've tried to go to AA, I just don't connect..for me, the medication changed my life.”

## Discussion

This is the first study to evaluate QG-A, an integrated telemedicine intervention combining behavioral and pharmacological treatment for AUD. Initial findings from this pilot investigation are promising, suggesting that delivery of CBT in conjunction with NTX through a virtual care platform is feasible and perceived as helpful. Likewise, most of the participants were retained in the digital intervention, engagement and completion rates were promising, and participants evidenced clinically meaningful changes in alcohol use. Moreover, though the QG-A intervention content did not address psychiatric symptoms directly, reductions in depression and anxiety, coupled with increased resiliency, are encouraging.

Given the low rates of treatment utilization among those with AUD, along with the rise in problematic alcohol use corresponding with COVID-19, innovations in telemedicine and m-health have the potential to be transformative, expanding access to evidence-based therapies. Likewise, several professional health organizations and national agencies recommend leveraging telemedicine to expand the availability of addiction medicine expertise, particularly among populations with limited access to specialty treatment.^15^

Though AUD populations are often difficult to engage and retain in care, preliminary evidence gathered in this study suggests that an m-health approach can overcome these challenges. Although recent efforts to expand telemedicine for addictions have concentrated on medications for opioid use disorders, the findings from this study suggest that broadening access to evidence-based behavioral and pharmacological treatment for AUD through telehealth is feasible and potentially efficacious.

Despite the promise of our preliminary outcomes, several limitations of the present investigation are noteworthy. This study included a small sample size, a self-selected group, and absence of a comparison group and longer-term follow-up. In addition, the extent of participants' prior exposure to CBT and NTX was not assessed and could have impacted their treatment response.

Nevertheless, given extensive evidence for the efficacy of the intervention approaches used (i.e., CBT and NTX),^3^ the changes observed are likely at least partially attributable to QG-A, suggesting that transporting these approaches to an m-health platform is feasible, acceptable, and potentially efficacious. A planned fully powered randomized trial will address many of the limitations of this pilot investigation.

## Abbreviations Used

AUD: alcohol use disorder
CBT: cognitive behavioral therapy
MET: motivational enhancement therapy
MM: medication management
NTX: naltrexone
QG-A: Quit Genius for AUD
SD: standard deviation
WHO: World Health Organization
